# Symmetry Based Automatic Evolution of Clusters: A New Approach to Data Clustering

**DOI:** 10.1155/2015/796276

**Published:** 2015-08-03

**Authors:** Singh Vijendra, Sahoo Laxman

**Affiliations:** ^1^Department of Computer Science and Engineering, Faculty of Engineering and Technology, Mody University of Science and Technology, Lakshmangarh, Rajasthan 332311, India; ^2^School of Computer Engineering, KIIT University, Bhubaneswar 751024, India

## Abstract

We present a multiobjective genetic clustering approach, in which data points are assigned to clusters based on new line symmetry distance. The proposed algorithm is called multiobjective line symmetry based genetic clustering (MOLGC). Two objective functions, first the Davies-Bouldin (DB) index and second the line symmetry distance based objective functions, are used. The proposed algorithm evolves near-optimal clustering solutions using multiple clustering criteria, without a priori knowledge of the actual number of clusters. The multiple randomized *K* dimensional (*Kd*) trees based nearest neighbor search is used to reduce the complexity of finding the closest symmetric points. Experimental results based on several artificial and real data sets show that proposed clustering algorithm can obtain optimal clustering solutions in terms of different cluster quality measures in comparison to existing SBKM and MOCK clustering algorithms.

## 1. Introduction

Clustering is one of the most common unsupervised data mining methods to explore the hidden structures embedded in a data set [1]. Clustering gives rise to a variety of information granules whose use reveals the structure of data [2]. Clustering has been effectively applied in a variety of engineering and scientific disciplines [3]. In order to mathematically identify clusters in a data set, it is usually necessary to first define a measure of similarity or proximity which will establish a rule for assigning patterns to the domain of a particular cluster center. Symmetry is considered as a preattentive feature that enhances recognition and reconstruction of shapes and objects. However, the exact mathematical definition of symmetry, such as Miller [4], is inadequate to describe and quantify symmetry found in the natural world or those found in the visual world. Since symmetry is so common in the abstract and in the nature, it is reasonable to assume that some kind of symmetry occur in the structures of clusters. The immediate problem is how to find a way to measure symmetry. Zabrodsky et al. [5] have proposed a kind of symmetry distance to detect symmetry in a figure extracted from an image. Their basic strategy is to choose the symmetry that is the closest to the figure measured by an appropriate measure, in which they adopt the minimum sum of the squared distances over which the vertices must be removed to impose the assumed symmetry. It follows that we need an algorithm for effectively imposing a given symmetry with a minimum displacement [6]. A new type of nonmetric distance, based on point symmetry, is proposed which is used in a *K*-means based clustering algorithm, referred to as symmetry based *K*-means (SBKM) algorithm [7]. SBKM will fail for some data sets where the clusters themselves are symmetrical with respect to some intermediate point. This work is extended in Chung and Lin [8] to overcome some of limitations existing in SBKM. These symmetry based clustering techniques adopted the concept of *K*-means for discovering clusters. The *K*-means [9] algorithm is one of the more widely used clustering algorithms. However, it is well known that *K*-means algorithm is sensitive to the initial cluster centers and easy to get stuck at the local optimal solutions. Second important problem in partitioning clustering is to find a partition of the given data, with a specified number of clusters that minimizes the total within cluster variation. Unfortunately in many real life cases the number of clusters in a data set is not known a priori.

In order to overcome the limitation of being easy to get stuck at the local optimal solutions, some attempts have been made to use genetic algorithms for clustering data sets [10–12]. To overcome the problem of automatic cluster determination from the data sets. Recently, many automatic clustering techniques have been introduced. These automatic clustering techniques are based on genetic algorithm methods and Differential Evolution (DE) methods. A fuzzy variable string length based point symmetry genetic clustering technique is proposed in [13]. It automatically evolves the appropriate types of clusters, both convex and nonconvex, which have some symmetrical structures. It fails if the clusters do not have symmetry property. In [14], a two-stage genetic clustering algorithm (TGCA) is proposed. It can automatically determine the proper number of clusters and the proper partition from a given data set. It is suitable for clustering the data with compact spherical clusters only. Single objective genetic clustering methods [15] fail to solve the issues of clusters shape and size simultaneously. They are suffering from ineffective genetic search, which in turn get stuck at suboptimal clustering solutions [16]. To overcome the limitations of these algorithms some attempts have been made to use multiobjective genetic algorithms. Handl and Knowles [17] proposed multiobjective clustering with automatic *K*-determination (MOCK) to detect the optimal number of clusters from data sets. But due to the heuristic nature of the algorithm, it provides an approximation to the real (unknown) Pareto front only. Therefore, generation of the best clustering solution cannot be guaranteed, and result shows some variation between individual runs. Saha and Bandyopadhyay [18] proposed a multiobjective clustering technique. In this algorithm points are assigned to different clusters based on the point symmetry based distance. It is able to detect clusters having point symmetry property. However it will fail for clusters having nonsymmetrical shape. Some researchers have applied Differential Evolution (DE) to the task of automatic clustering from data sets. In [19] a Differential Evolution (DE) technique on the point symmetry based cluster validity index is presented. To find the optimal number of clusters, they proposed a modified symmetry based index. The main limitation of this algorithm is problem-dependent dynamic control factor. Suresh et al. [20, 21] applied the Differential Evolution (DE) to the task of automatic fuzzy clustering, where two conflicting fuzzy validity indices are simultaneously optimized. They used a real-coded representation of the search variables to accommodate variable number of cluster centers [20, 21]. It depends on cluster centroid and thus is biased in any sense towards spherical clusters. Tremendous research effort has gone in the past few years to evolve the clusters in complex data sets through evolutionary techniques. Most clustering algorithms assume the number of clusters to be known a priori. The desired granularity [22] is generally determined by external, problem criteria. There seems to be no definite answer to how many clusters are in data set a user defined criterion for the resolution has to be given instead. Second, most of the existing clustering algorithms adopt 2-norm distance measures in the clustering. These measures fail when clusters tend to develop along principal axes. The symmetry based clustering techniques also seek for clusters which are symmetric with respect to their centers. Thus, these techniques will fail if the clusters do not have this property.

The objective of this paper is twofold. First, it aims at the automatic determination of the optimal number of clusters in any data set. Second, it attempts to find clusters of arbitrary shapes and sizes. We show that genetic algorithm with a new line symmetry based distance can give very promising results if applied to the automatic clustering problem. The proposed algorithm evolves near-optimal clustering solutions using multiple clustering criteria, without a priori knowledge of the actual number of clusters. The multiple randomized* Kd* trees based nearest neighbor search is used to reduce the complexity of finding the closest symmetrical points. We refer to this new algorithm as the multiobjective line symmetry based genetic clustering (MOLGC) algorithm. We have compared the MOLGC with two other clustering techniques: SBKM and MOCK. The following performance metrics have been used in the comparative analysis: (1) the accuracy of final clustering results; (2) the computation time; and (3) the statistical significance test.

This paper is organized as follows: The related work on symmetry is reviewed in Section 2. In Section 3, proposed line symmetry measure with multiple randomized* Kd* trees based nearest neighbor search approach is presented. Multiobjective line symmetry based genetic clustering technique is explained in Section 4.  Section 5 contains data description and experimental results. Finally, we conclude in Section 6.

## 2. Related Work

In this section at first, point symmetry based distance is described in brief. Then line symmetry based distance is discussed.

### 2.1. Point Symmetry Based Distance

Symmetry is considered as a preattentive feature that enhances recognition and reconstruction of shapes and objects. Su and Chou [7] presented an efficient point symmetry distance (PSD) measure to help partitioning the data set into the clusters, where each cluster has the point symmetry property. The point symmetry distance measure between the data point *x*_*i*_, {*x*_*i*_∣for  1 ≤ *i* ≤ *N*}, and the data point *x*_*j*_ relative to the cluster centroid *c*_*k*_, {*c*_*k*_∣for  1 ≤ *k* ≤ *K*}, is defined as

(1) $$\begin{array}{c} d_{s}\left(x_{j},c_{k}\right)=\underset{\forall i\neq j,1\leq j\leq N}{\min}\frac{\left\|\left(x_{j}-c_{k}\right)+\left(x_{i}-c_{k}\right)\right\|}{\left\|\left(x_{j}-c_{k}\right)\right\|+\left\|\left(x_{i}-c_{k}\right)\right\|}, \end{array}$$

for *i* ≠ *j* and 1 ≤ *i* ≤ *N*, where ‖·‖ denotes the 2-norm distance. Note that distance *d*_*s*_(*x*_*j*_, *c*_*k*_) is minimized when the pattern *x*_*i*_ = (2 × *c*_*k*_ − *x*_*j*_) exists in the data sets. They have proposed a symmetry based *K*-means clustering algorithm called SBKM which assigns the patterns to a particular cluster depending on the symmetry based distance *d*_*s*_ only when *d*_*s*_ is greater than some user specified threshold *θ* = 0.18. Otherwise, assignment is done according to the Euclidean distance. We can demonstrate how the point symmetry distance (PSD) measure works well for the case of symmetrical intra-clusters. Assume positions of two centroids *c*_1_ and *c*_2_ are *c*_1_ = (16,10) and *c*_2_ = (16,19). The *x*_1_, *x*_2_, and *x*_3_ are three data points and their positions are *x*_1_ = (14,16), *x*_2_ = (18,4), and *x*_3_ = (19,25), respectively. The PSD measure between data points *x*_1_ and cluster center *c*_1_ is

(2)  dsx1,c1=x1−c1+x2−c1x1−c1+x2−c1=040+40=0, dsx1,c2=x1−c2+x3−c2x1−c2+x3−c2=1013+45=0.30.

**Figure 1:**
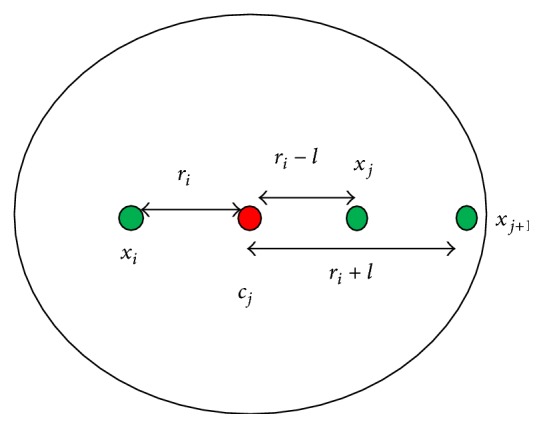
An example for the distance difference symmetry.

Because *d*_*s*_(*x*_1_, *c*_1_) < *d*_*s*_(*x*_1_, *c*_2_) and *d*_*s*_(*x*_1_, *c*_1_) is less than the specified threshold (0.18), the data point *x*_2_ is said to be the most symmetrical point of *x*_1_ relative to *c*_1_; thus we have *x*_2_ = Arg *d*_*s*_(*x*_1_, *c*_1_). Consequently, assigning the data point *x*_1_ to the cluster *C*_1_ is a good decision. But the problems occurring in the point symmetry distance (PSD) measure are (1) lacking the distance difference symmetry property, (2) leading to an unsatisfactory clustering result for the case of symmetrical inter-clusters. In the first problem, the PSD measure favors the far data point when we have more than two symmetrical data points and this may degrade the symmetrical robustness. We can depict this problem as shown in Figure 1.

Let *x*_*i*_ = (−5,0), *x*_*j*_ = (7,0), *x*_*j*+1_ = (10,0), and *c*_*j*_ = (0,0); then find the most symmetry point of *x*_*i*_ relative to *x*_*j*_ and *x*_*j*+1_,

(3) dsxi,cj=min⁡xi−cj+xj−cjxi−cj+xj−cj,    xi−cj+xj+1−cjxi−cj+xj+1−cj,dsxi,cj=min⁡212,5125=min⁡0.16,0.04.

The data point *x*_*j*+1_ is selected as the most symmetrical point of *x*_*i*_ relative to the centroid *c*_*j*_. This shows that (1) favors the far data point when we have more than two data points and this may corrupt the symmetrical robustness.

**Figure 2:**
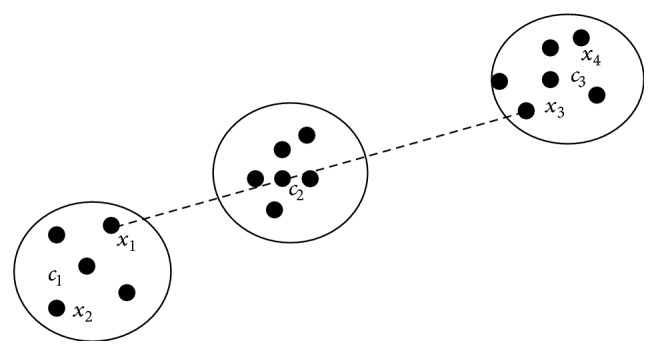
Point symmetry interclusters distance.

In the second problem, if two clusters are symmetrical to each other with respect to the centroid of any third cluster, then PSD measure gives an unsatisfactory clustering result. As presented in Figure 2, the cluster *C*_1_ and the cluster *C*_3_ are symmetrical to the cluster center *c*_2_.

Let *x*_1_ = (−10, −2), *x*_2_ = (−12, −4), *x*_3_ = (12,2), *x*_4_ = (13,4), *c*_1_ = (−11, −3), *c*_2_ = (0,0), and *c*_3_ = (12,3); then for the data point *x*_1_, and by (1), we have

(4) dsx1,c1=x1−c1+x2−c1x1−c1+x2−c1=82+50=0.94,dsx1,c2=x1−c2+x3−c2x1−c2+x3−c2=2104+148=0.08,dsx1,c3=x1−c3+x4−c3x1−c3+x4−c2=457509+2=0.89.

**Figure 3:**
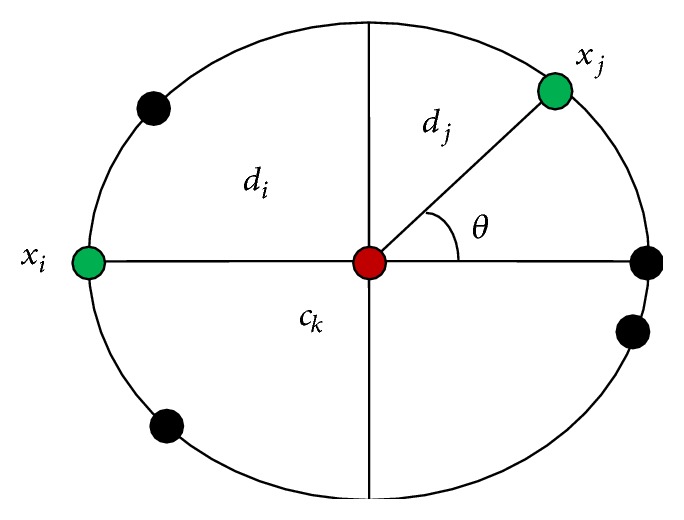
An example for distance difference.

In the above example point symmetry distance *d*_*s*_(*x*_1_,*c*_2_) < *d*_*s*_(*x*_1_,*c*_3_) < *d*_*s*_(*x*_1_,*c*_1_) is smallest among all distances, so the data point *x*_1_ should be assigned to the cluster *C*_2_, but it conflicts our visual assessment. Due to the above two problems, Chung and Lin [8] proposed a symmetry based distance measure known as Symmetry Similarity Level (SSL), which satisfies the distance difference symmetry property. Let *c*_*k*_ denote the cluster centroid; *x*_*i*_ and *x*_*j*_ denote two related data points as shown in Figure 3.

Let $d_{i}=\bar{x_{i}c_{k}}$ and $d_{j}=\bar{x_{j}c_{k}}$; then the Distance Similarity Level (DSL) operator for measuring the distance difference symmetry between the distance $\bar{x_{i}c_{k}}$ and the distance $\bar{x_{j}c_{k}}$ is defined by

(5) $$\begin{array}{c} \text{DSL}\left(x_{i},c_{k}{,x}_{j}\right)=\left\{\begin{array}{cc} 1-\frac{\left|d_{i}-d_{j}\right|}{d_{i}}, & \text{if}\;0\leq \frac{d_{j}}{d_{i}}\leq 2\\ 0 & \text{otherwise}. \end{array}\right. \end{array}$$

They replaced the interval 0 ≤ *d*_*j*_/*d*_*i*_ ≤ 2 to the interval 0 ≤ *d*_*j*_/*d*_*i*_ ≤ *k*, *k* > 2; in (5), the number of examined symmetrical points will be increased and the computational gain might be degraded. They proposed second component called Orientation Similarity Level (OSL). By applying the projection concept, the OSL between the two vectors, $v_{i}=\vec{x_{i}c_{k}}=\left(c_{k}-x_{i}\right)$ and $v_{j}=\vec{c_{k}x_{j}}=(x_{j}-c_{k})$, is defined by

(6) $$\begin{array}{c} \text{OSL}\left(x_{i},c_{k}{,x}_{j}\right)=\frac{v_{i}\cdot v_{j}}{2\left\|v_{i}\right\|\left\|v_{j}\right\|}+0.5. \end{array}$$

By (5) and (6), they combined the effect of DSL(*x*_*i*_, *c*_*k*_,*x*_*j*_) and OSL(*x*_*i*_, *c*_*k*_,*x*_*j*_) to define a Symmetry Similarity Level (SSL) between the vectors $\vec{x_{i}c_{k}}$ and $\vec{c_{k}x_{j}}$. They defined the following:

(7) $$\begin{array}{c} \text{SSL}\left(x_{i},c_{k}{,x}_{j}\right)=\sqrt{\frac{{\text{DSL}}^{2}\left(x_{i},c_{k},x_{j}\right)+{\text{OSL}}^{2}\left(x_{i},c_{k},x_{j}\right)}{2}}, \end{array}$$

for 1 ≤ *k* ≤ *K* and 1 ≤ *i* ≤ *N*. Because of 0 ≤ DSL(*x*_*i*_, *c*_*k*_,*x*_*j*_) ≤ 1 and 0 ≤ OSL(*x*_*i*_, *c*_*k*_, *x*_*j*_) ≤ 1, it is easy to verify that 0 ≤ SSL(*x*_*i*_, *c*_*k*_,*x*_*j*_) ≤ 1 is held. Based on SSL operator, Chung and Lin [8] proposed a modified point symmetry based *K*-means (MPSK) algorithm. The time complexity for finding the symmetry point for *n* objects is *O* (*kn* ^2^). So this approach is not suitable for large and high dimensional data sets. To overcome limitations of SBKM, Bandyopadhyay and Saha [23] proposed new point symmetry based clustering algorithm known as variable string length genetic clustering technique with point symmetry (VGAPS). The proposed point symmetry distance is defined as follows. The symmetrical (reflected) point of *x* with respect to a particular centre *c* is 2 × *c* − *x* that is denoted by *x* ^*^. Let *k* unique nearest neighbors of *x* ^*^ be at Euclidean distances of *d*_*i*_, *i* = 1,2,…, *k*. Then *d*_ps_(*x*, *c*) = *d*_*sym*_(*x*, *c*) × *d*_*e*_(*x*, *c*):

(8) $$\begin{array}{c} d_{\text{ps}}\left(x,c\right)=d_{\mathrm{sym}}\left(x,c\right)\times d_{e}\left(x,c\right)=\frac{\sum_{i=1}^{k}d_{i}}{k}\times d_{e}\left(x,c\right), \end{array}$$

where *d*_*e*_(*x*, *c*) is the Euclidean distance between the point *x* and cluster center *c*. It can be seen from (8) that *k* cannot be chosen equal to 1, since if point *x* ^*^ exists in the data set then *d*_ps_(*x*, *c*) = 0 and hence there will be no impact of the Euclidean distance. To overcome this problem, they have taken average distance between reflected point *x* ^*^ and its first and the second unique nearest neighbor's points. They proposed a rough guideline of the choice of *θ*, the threshold value on the point symmetry distance that is equal to maximum nearest neighbor distance *d*_NN_^max⁡^ in the data set. For reducing the complexity of point symmetry distance computation,* Kd* tree based data structure is used. VGAPS detects clusters which are point symmetry with respect to their centers. Thus VGAPS will fail if the clusters do not have this property.

### 2.2. Existing Line Symmetry Based Distance

From the geometrical symmetry view point, point symmetry and line symmetry are two widely discussed issues. Motivation by this, Saha and Maulik [24] proposed a new line symmetry based automatic genetic clustering technique called variable string length genetic line symmetry distance based clustering (VGALS-Clustering). To measure amount of line symmetry of a point *x* with respect to a particular line *i*, *d*_ls_(*x*, *i*), the following steps are followed:

(1) For a particular data point *x*, calculate the projected point *p*_*i*_ on the relevant symmetrical line *i*.
(2) Find *d*_*sym*_(*x*,*p*_*i*_) as
  (9) $$\begin{array}{c} d_{\mathrm{sym}}\left(x{,p}_{i}\right)=\frac{\sum_{i=1}^{k}d_{i}}{k}, \end{array}$$
  where *k* nearest neighbors of *x* ^*^ = 2 × *p*_*i*_ − *x* are at Euclidean distances of *d*_*i*_, *i* = 1,2,…, *k*. Then the amount of line symmetry of a particular point *x* with respect to that particular symmetrical line of cluster *i* is calculated as
  (10) $$\begin{array}{c} d_{\text{ls}}\left(x,i\right)=d_{\mathrm{sym}}\left(x,p_{i}\right)\times d_{e}\left(x,c\right), \end{array}$$
  where *c* is the centroids of the particular cluster *i* and *d*_*e*_(*x*, *c*) is the Euclidean distance between the points *x* and *c*. The possible problem existing in this given line symmetry measure is lacking the closure property. The closure property can be expressed as follows: if the data point *p*_*i*_ is currently assigned to the cluster centroid *c*_*k*_ in the current iteration, the determined most symmetrical point *p*_*j*_ relative to *c*_*k*_ must have been assigned to *c*_*k*_ in the previous iteration.

## 3. Proposed Line Symmetry Measure

**Figure 4:**
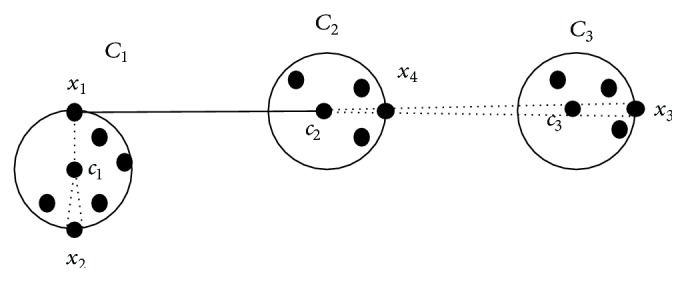
Violation of closure property.

Both point symmetry and line symmetry distance lack the closure property and this would result in an unsatisfactory clustering result. According to the symmetry property, the data point *x*_1_ in Figure 4, which is not in the cluster *C*_2_ originally, if symmetry distance *d*_*sym*_(*x*_1_,*c*_2_) of point *x*_1_ with cluster center *c*_2_ is the most symmetrical distance (*d*_*sym*_(*x*_1_,*c*_2_) < *d*_*sym*_(*x*_1_,*c*_1_) < *d*_*sym*_(*x*_1_,*c*_3_)) among other symmetry distances, it tells us that data point *x*_1_ should currently be assigned to the cluster *C*_2_. But the most symmetrical point of *x*_1_ relative to the centroid *c*_2_ is the data point *x*_3_, which has been assigned to the centroid *c*_3_. Since the data point *x*_3_ has not been assigned to the centroid *c*_2_ before, it violates closure property. It would give an unsatisfactory clustering result.

**Figure 5:**
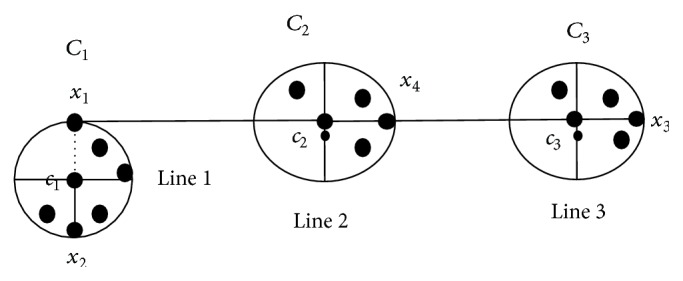
An example of proposed line symmetry distance.

**Figure 6:**
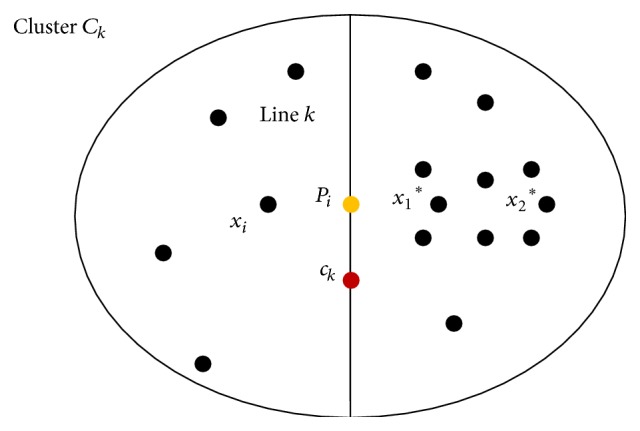
An example of two symmetry points.

By considering above problem in existing symmetry based distances, we have applied a constraint in new line symmetry distance measure to satisfy closure property, in which, to compute the line symmetry distance of the data point *x*_*i*_, we have restricted the candidate symmetrical points *x*_*j*_ ∉ *C*_*k*_ relative to each symmetrical line *k* of the corresponding cluster *C*_*k*_. For the data point *x*_*i*_ relative to symmetrical line of cluster *C*_*k*_, this restriction can help us to search more suitable symmetrical point *x*_*j*_, because we ignore the candidate most symmetrical point *x*_*j*_ which is not in the cluster *C*_*k*_. As depicted in Figure 5, let the point *x*_1_ have most line symmetry distance (*d*_ls_(*x*_1_,*C*_2_) < *d*_ls_(*x*_1_,*C*_1_) < *d*_ls_(*x*_1_,*C*_3_)) with respect to particular line of cluster *C*_2_ and the symmetrical point is *x*_3_, but due to the above constraint the proposed line symmetry distance method is assigned the point *x*_1_ to cluster *C*_1_. The assignment of *x*_1_ to the cluster *C*_1_ is a reasonable assignment from our visual system. We applied the second modification in which the first and second symmetrical points *x*_1_^*^ and *x*_2_^*^ of point *x*_*i*_ are found in cluster *C*_*k*_ (as shown in Figure 6) relative to the symmetrical line, not in all data points; that is, each point *x*_*i*_, 1 ≤ *i* ≤ *n*, is assigned to cluster *C*_*k*_ iff *d*_ls_(*x*_*i*_,*C*_*k*_) ≤ *d*_ls_(*x*_*i*_,*C*_*j*_), where *j*, *k* = 1,…, *k* and *j* ≠ *k*, *d*_ls_(*x*_*i*_,*C*_*k*_)/*d*_*e*_(*x*_*i*_,*c*_*k*_) ≤ *θ*, and *x*_1_^*^ and *x*_2_^*^ belong to cluster *C*_*k*_. The distance *d*_ls_(*x*_*i*_,*C*_*k*_) is calculated as given in (22) and *θ* = *d*_nn_^max⁡^ is the symmetrical threshold, where *d*_nn_^max⁡^ = max⁡_*i*=1,…,*n*_ *d*_nn_(*x*_*i*_) and the distance *d*_nn_(*x*_*i*_) is the maximum nearest neighbor distance in the data set.

The value of *θ* is kept equal to the maximum nearest neighbor distance among all the points in the data set. Point assignment based on proposed line symmetry distance is given in Algorithm 1.

For computing the proposed line symmetry distance of a given data set, we find the symmetrical line of each cluster by using central moment method [25] that is used to measure the Symmetry Similarity Level between two data points relative to symmetrical line. Let the data set be denoted by *X* = {(*x*_1_, *y*_1_), (*x*_2_, *y*_2_), (*x*_*n*_, *y*_*n*_)}; then the (*p*, *q*)th order moment is defined as

(11) $$\begin{array}{c} m_{pq}=\underset{\forall (x_{i},y_{i})\in X}{\sum }x_{i}^{p}y_{i}^{q}. \end{array}$$

The centroid of the given data set for one cluster is defined as (*m*_10_/*m*_00_, *m*_01_/*m*_00_). The central moment is defined as

(12) $$\begin{array}{c} u_{pq}=\underset{\forall (x_{i},y_{i})\in X}{\sum }{\left(x_{i}-\overset{-}{x}\right)}^{p}{\left(y_{i}-\overset{-}{y}\right)}^{q}, \end{array}$$

where $\bar{x}=m_{10}/m_{00}$ and $\bar{y}=m_{01}/m_{00}$. According to the calculated centroid and (12), the major axis of each cluster can be determined by the following two items:

(a) The major axis of the cluster must pass through the centroids.
(b) The angle between the major axis and the *x*-axis is equal to (1/2)tan^−1^(2*u*_11_/(*u*_20_ − *u*_02_)).

Consequently, for one cluster, its corresponding major axis is thus expressed by

(13) $$\begin{array}{c} \left(\left(\frac{m_{10}}{m_{00}},\frac{m_{01}}{m_{00}}\right),\frac{1}{2}\tan^{-1}\left(\frac{2u_{11}}{u_{20}-u_{02}}\right)\right). \end{array}$$

**Figure 7:**
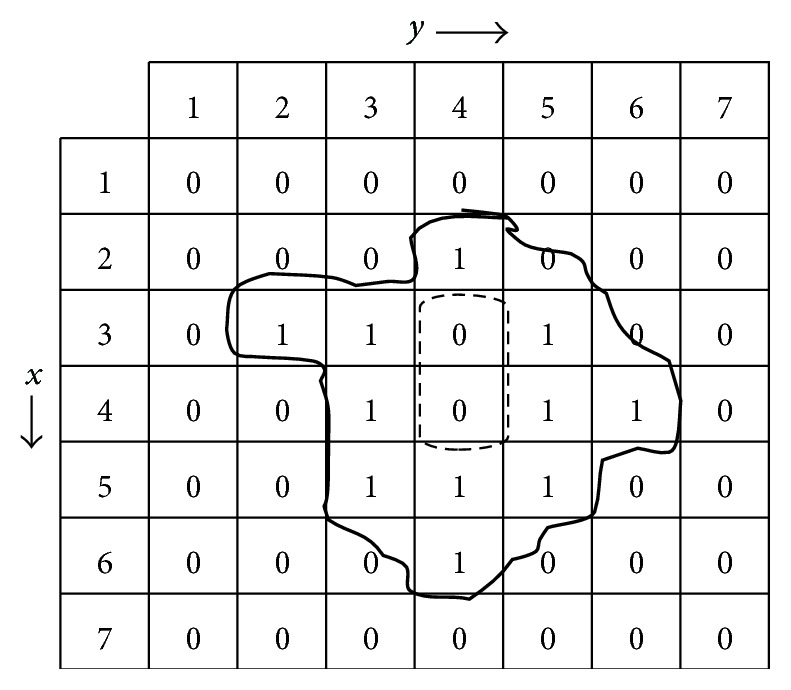
A region *R* in binary image.

Let normalized form of data points be stored into memory of the computer system. Now we can apply central moment method for computing the shape of the data points. A brief mathematical calculation for finding the symmetrical line of each cluster by using central moment method is given in Figure 7 and below:

(14)  m00=∑∀(xi,yi)∈Xxi0yi0=Area=11, m01=∑∀(xi,yi)∈Xxi0yi1=44, m10=∑∀(xi,yi)∈Xxi1yi0=44, m11=∑∀(xi,yi)∈Xxi1yi1=178, m02=∑∀(xi,yi)∈Xxi0yi2=190, m20=∑∀(xi,yi)∈Xxi2yi0=190.

The centroid of cluster is calculated as

(15) $$\begin{array}{c} \bar{x}=\frac{m_{10}}{m_{00}}=\frac{44}{11}=4,\;\;\bar{y}=\frac{m_{01}}{m_{00}}=\frac{44}{11}=4. \end{array}$$

We can apply centroid $\left(\bar{x},\bar{y}\right)=(4,4)$ of cluster for computing the central moments. The physical significance of the central moments is that they just give the area and the moment of inertia. The lower order central moments (Zero_th_) give the area of the region *R*:

(16) $$\begin{array}{c} u_{00}=\underset{\forall (x_{i},y_{i})\in X}{\sum }{\left(x_{i}-4\right)}^{0}{\left(y_{i}-4\right)}^{0}=11. \end{array}$$

The product moment involves finding the product of $\left(x_{i}-\overset{-}{x}\right)$ and $\left(y_{i}-\overset{-}{y}\right)$ increasing to a power

(17) $$\begin{array}{c} u_{11}=\underset{\forall (x_{i},y_{i})\in X}{\sum }{\left(x_{i}-4\right)}^{1}{\left(y_{i}-4\right)}^{1}=2. \end{array}$$

The second order central moment along *x*-axis is

(18) $$\begin{array}{c} u_{02}=\underset{\forall (x_{i},y_{i})\in X}{\sum }{\left(x_{i}-4\right)}^{0}{\left(y_{i}-4\right)}^{2}=14. \end{array}$$

The second order central moment along *y*-axis is

(19) $$\begin{array}{c} u_{20}=\underset{\forall (x_{i},y_{i})\in X}{\sum }{\left(x_{i}-4\right)}^{2}{\left(y_{i}-4\right)}^{0}=13. \end{array}$$

The angle between the major axis and the *x*-axis is

(20) $$\begin{array}{c} \frac{1}{2}\tan^{-1}\left(\frac{2u_{11}}{u_{20}-u_{02}}\right)=38^{o}. \end{array}$$

The obtained major axis is treated as the symmetric line of the relevant cluster. This symmetrical line is used to measure the amount of line symmetry of a particular point in that cluster. In order to measure the amount of line symmetry of a point (*x*_*i*_) with respect to a particular line *k* of cluster *C*_*k*_, *d*_ls_(*x*_*i*_,*C*_*k*_), the following steps are followed.

**Figure 8:**
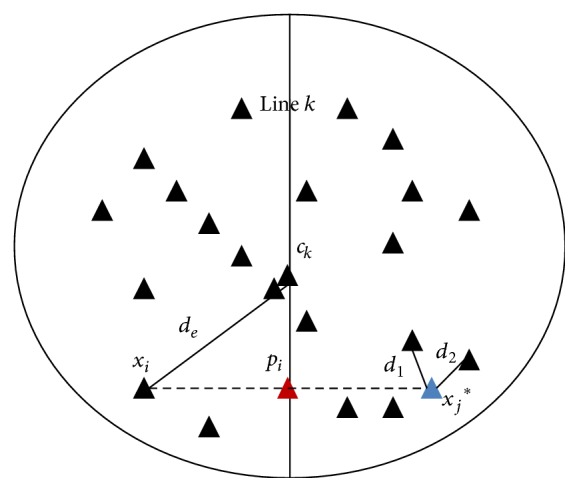
An example for computing line symmetry distance.

(1) For a particular data point *x*_*i*_, calculate the projected point *p*_*i*_ on the relevant symmetrical line *k* of cluster *C*_*k*_ (as shown in Figure 8) and then find out all possible symmetrical data point *x*_*j*_ relative to each symmetrical line *k* for 1 ≤ *i* ≤ *n*, 1 ≤ *j* ≤ *n*, and 1 ≤ *k* ≤ *K*.
(2) Find *d*_*sym*_(*x*_*i*_,*p*_*i*_) as
  (21) $$\begin{array}{c} d_{\mathrm{sym}}\left(x_{i}{,p}_{i}\right)=\frac{\sum_{i=1}^{k\text{near}}d_{i}}{k\text{near}}, \end{array}$$
  where *k* nearest neighbors of *x*_*j*_ = 2 × *p*_*i*_ − *x*_*i*_ are at Euclidean distances of *d*_*i*_, *i* = 1,2,…, *k*near. In fact, the role of the parameter *k*near is intuitively easy to understand and it can be set by the user based on specific knowledge of the application. In general, a fixed value of *k*near may have many drawbacks. For clusters with too few points, the points likely to be scattered and the distance between two neighbors may be too large. For very large cluster fixed number of neighbors may not be enough because few neighbors would have a distance close to zero. Obviously, the parameter *k*near is related to the expected minimum cluster size and should be much smaller than the number of objects in the data. To gain a clear idea of the distance of the neighborhood of a point, we have chosen $k\text{near}\leq \sqrt{n}$ in our implementation. The randomized* Kd* trees based nearest neighbor search is used to reduce the computation time of the nearest neighbors. The amount of line symmetry of a particular point *x*_*i*_ with respect to particular symmetrical line of cluster *C*_*k*_ is calculated as
  (22) $$\begin{array}{c} d_{\text{ls}}\left(x_{i},C_{k}\right)=d_{\mathrm{sym}}\left(x_{i},p_{i}\right)\times d_{e}\left(x_{i},c_{k}\right), \end{array}$$
  where *c*_*k*_ is the centroid of the cluster *C*_*k*_ and *d*_*e*_(*x*_*i*_, *c*_*k*_) denotes Euclidean distance between data point *x*_*i*_ and cluster center *c*_*k*_.

### 3.1. Multiple Randomized* Kd* Trees Based Nearest Neighbor Search

The problem of nearest neighbor search is one of major importance in a variety of applications such as image recognition, data compression, pattern recognition and classification, machine learning, document retrieval systems, statistics, and data analysis. The most widely used algorithm for nearest neighbor search is the *K* dimensional tree (*Kd* tree) [26–30]. This works well for exact nearest neighbor search in low dimensional data but quickly loses its effectiveness as dimensionality increases. In high dimensions to find the nearest neighbor may require searching a very large number of nodes. However, solving this problem in high dimensional spaces seems to be a very difficult task and there is no algorithm that performs significantly better than the standard brute-force search. To address this problem, Anan and Hartley [31] have investigated the following strategies: (1) They create *m* different* Kd* trees each with a different structure in such a way that searches in the different trees will be (largely) independent. (2) With a limit of *n* nodes to be searched, they break the search into simultaneous searches among all the *m* trees. On average, *n*/*m* nodes will be searched in each of the trees. (3) The principal component analysis is used to rotate the data to align its moment axes with the coordinate axes. Data will then be split up in the tree by hyperplanes perpendicular to the principal axes. They have written that either by using multiple search trees or by building the* Kd* tree from data realigned according to its principal axes, search performance improves and even improves further when both techniques are used together.

To overcome the above problem, we have used the approximate nearest neighbor search approach, in which the randomized trees are built by choosing the split dimension randomly from the first *d* dimensions on which data has the greatest variance and each tree is constructed independently [32]. In proposed MOLGC algorithm, instead of always splitting on the maximally variant dimension, each tree selects randomly among the top five most variant dimensions at each level. When searching the trees, a single priority queue is maintained across all the randomized trees so that search can be ordered by increasing distance to each bin boundary. The degree of approximation is determined by examining a fixed number of leaf nodes, at which point the search is terminated and the best candidates returned. In the multiple randomized* Kd* trees based nearest neighbor search technique, the data points *X* = *x*_1_, *x*_2_,…, *x*_*n*_ are preprocessed into a metric space *S*, so that, given any query point *q* ∈ *X*, the nearest or generally *k* nearest points of *x* to *q* can be reported efficiently. In proposed MOLGC algorithm to find line symmetric distance of a particular point *x*_*i*_ with respect to the symmetrical line of cluster *C*_*k*_, we have to find the nearest neighbors of *x*_*j*_ (where *x*_*j*_ = 2 × *p*_*i*_ − *x*_*i*_ for 1 ≤ *i* ≤ *n* and 1 ≤ *j* ≤ *n*). Therefore the query point *q* is set equal to *x*_*j*_. After getting the *k* nearest neighbors of *x*_*j*_, the line symmetrical distance of *x*_*i*_ to the symmetrical line of cluster *C*_*k*_ is calculated by using (22).

## 4. Multiobjective Line Symmetry Based Genetic Clustering Technique

In this section, a multiobjective genetic clustering technique using the proposed line symmetry based distance is proposed. The algorithm is known as multiobjective line symmetry based genetic clustering (MOLGC). The subsections of this section are organized as follows.

### 4.1. Chromosome Representation

In proposed algorithm, the numerical feature values of all cluster centers are encoded into a real coded chromosome as a clustering solution. The length of a particular chromosome *c* is *l*_*c*_, given by *l*_*c*_ = *d* × *K*, where *d* is the dimension of the data set and *K* is the number of cluster centers encoded in that chromosome.

For example a chromosome representation (5.5 3.5 4.2 4.0 2.5 3.5 6.2 7.3 1.5 2.5 3.5 4.5) has three cluster centers in four dimensional feature space. The encoded three clusters are (5.5 3.5 4.2 4.0), (2.5 3.5 6.2 7.3), and (1.5 2.5 3.5 4.5). For a variable-length chromosome representation, each chromosome has the initial length *l*_*c*_. The number of clusters, denoted by *K*, is randomly generated in the range [*K*_min⁡_, *K*_max⁡_]. Here *K*_min⁡_ is chosen as 2, and *K*_max⁡_ is chosen to be $\sqrt{n}$, where *n* is the size of the data set. Their after *K*-means algorithm is executed with the set of centers encoded in each chromosome. The resultant centers are used for replacing the centers in the corresponding chromosomes. The steps of proposed MOLGC algorithm are given in Algorithm 2.

### 4.2. Fitness Computation

The fitness of an individual indicates the degree of suitability of the solution it represents. In general, the fitness of a chromosome is evaluated using the objective function of the problem. The first objective of the clustering problem considered in this paper is to maximize the similarity within each cluster and the dissimilarity among clusters. The second objective is to detect clusters based on line symmetry distance. In this paper, two objective functions, the Davies-Bouldin (DB) index [33] and proposed line symmetry distance, are used to evaluate the fitness of a chromosome. The DB index is used to find clusters which are compact and well separated by minimizing the intracluster distance while maximizing the intercluster distance. DB index is the ratio of the sum of within cluster scatter *S*_*i*,*q*_ of cluster *C*_*i*_ to between cluster separations. Within cluster scatter *S*_*i*,*q*_ of cluster *C*_*i*_ is defined as

(23) $$\begin{array}{c} S_{i,q}={\left(\frac{1}{C_{i}}\underset{x\in C_{i}}{\sum }{\left\|x-c_{i}\right\|}^{q/2}\right)}^{1/q}, \end{array}$$

where *c*_*i*_ denotes the cluster center of cluster *C*_*i*_. Cluster center *c*_*i*_ is computed as

(24) $$\begin{array}{c} c_{i}=\frac{1}{n_{i}}\underset{x\in C_{i}}{\sum }x, \end{array}$$

where *n*_*i*_ denotes the number of the objects belonging to cluster *C*_*i*_. The within cluster scatter *S*_*i*,*q*_ denotes the *q*th root of the *q*th moment of the objects belonging to cluster *C*_*i*_ with respect to their mean. The distance between clusters *C*_*i*_ and *C*_*j*_ is denoted as *d*_*ij*_ and is defined as *d*_*ij*_ = *d*_*e*_(*c*_*i*_, *c*_*j*_), where *d*_*e*_ stands for Euclidean distance between the centroids *c*_*i*_ and *c*_*j*_ of the clusters *C*_*i*_ and *C*_*j*_, respectively. Then, DB index is defined as

(25) $$\begin{array}{c} \text{DB}=\frac{1}{k}\overset{k}{\underset{i=1}{\sum }}R_{i,q}. \end{array}$$

Here,

(26) $$\begin{array}{c} R_{i,q}=\underset{i,i\neq j}{\max}\left\{\frac{S_{i,q}+S_{j,q}}{d_{ij}}\right\}, \end{array}$$

where *k* corresponds to the number of selected clusters. An individual cluster index is taken as the maximum pairwise comparison computed as the ratio of the sum of within cluster dispersions from the two partitions divided by a measure of the between cluster separation. Smaller values for DB index correspond to good clusters. We set the fitness *F*_*i*_ of chromosome *i* to be equal to 1/DB_*i*_, where DB_*i*_ is the DB index of individual *i*. The second objective function is based on proposed line symmetry distance. The procedure of the fitness computation is given in Algorithm 3.

The fitness function of the chromosomes fit_ls_ is defined as the inverse of *f*; that is,

(27) $$\begin{array}{c} {\text{fit}}_{\text{ls}}=\frac{1}{f}. \end{array}$$

### 4.3. Genetic Operators

In this subsection, genetic operators used in proposed clustering algorithm are discussed. These genetic operators pass genetic information between subsequent generations of the population.

#### 4.3.1. Selection

Pareto based selection is used to select fitter solutions in each step of the evolution. It is a stochastic selection method where the selection probability of a chromosome is proportional to the value of its fitness [34]. The fitness for a chromosome chrom, denoted by fitness (chrom), is converted from its Pareto count (or dominance count) of *x* in the whole population. The probability that the individual chrom is selected from the population is denoted by

(28) $$\begin{array}{c} p_{r}\left(i\right)=\frac{\text{fitness}\left(\text{chrom}\right)}{\sum_{i=1}^{P}{\text{chrom}}_{i}}, \end{array}$$

where *P* is the population size; comparing with the conventional roulette wheel selection method that is directly based on the fitness of solutions, Pareto-dominance based selection method can lower the selection pressure and increase the chances of the subspaces with low fitness to be selected into next population.

#### 4.3.2. Crossover

Crossover is a probabilistic process that exchanges information between two parent chromosomes for generating two child chromosomes [34]. For chromosomes of length *l*_*c*_, a random integer, called the crossover point, is generated in the range [1, *l*_*c*_ − 1]. The portions of the chromosomes lying to the right of the crossover point are exchanged to produce two offspring. Let parent chromosomes *C*_1_ and *C*_2_ encode *k*_1_ and *k*_2_ cluster centers, respectively. *l*_1_, the crossover point in *C*_1_, is generated as *l*_1_ = rand() mod *k*_1_. Let *l*_2_ be the crossover point in *C*_2_, and it may vary in between [LB(*k*_2_), RB(*k*_2_)], where LB() and RB() indicate the left and right bounds of the range of *k*_2_, respectively. LB(*k*_2_) and RB(*k*_2_) are given by

(29)  LB(k2)=min⁡[2,max⁡[0,2−(k1−l1)]], RB(k2)=[k2−max⁡[0,2−l1]].

Therefore *l*_2_ is given by

(30) $$\begin{array}{c} l_{2}=\left\{\begin{array}{cc} \text{LB}\left(l_{2}\right)+\text{rand}\left(\right)\mathrm{mod}\left(\text{RB}\left(l_{2}\right)-\text{LB}\left(l_{2}\right)\right), & \\ \;\;\;\;\;\;\;\text{if}\;\;\text{RB}\left(l_{2}\right)\geq \text{LB}\left(l_{2}\right) & \\ 0\;\;\;\;\;\;\;\text{otherwise}. & \end{array}\right. \end{array}$$

As an example, let two chromosomes *C*_1_  (10 20 15 25) and *C*_2_  (15 30 18 32 19 35 25 45 30 50) be with number of 2 and 5 clusters. Now we can apply crossover operation on *C*_1_ and *C*_2_ as

(31)  C1=10 20 15 25, C2=(15 30 18 32 19 35 25 45 30 50).

The crossover point in *C*_1_ is generated as

(32) $$\begin{array}{c} l_{1}=5\mathrm{mod}2=1, \end{array}$$

where 5 is random number generated by rand() function. The crossover point in *C*_2_ varies in between LB(*l*_2_) = min⁡[2, max⁡[0,2 − (2 − 1)]] = 1 and RB(*l*_2_) = [5 − max⁡[0,2 − 1]] = 4.

The crossover point in *C*_2_ is *l*_2_ = LB(*l*_2_) + rand() mod (RB(*l*_2_) − LB(*l*_2_)) = 1 + 5mod⁡(4 − 1) = 1 + 2 = 3

(33) l1                C1(10 20 ∣ 15 25)    l2          C215 30 18 32 19 35 ∣ 25 45 30 50. 

The offspring *C*_3_ and *C*_4_ generated after crossover operation are

(34) Offspring  C310 20 25 45 30 50,    Offspring  C415 30 18 32 19 35 15 25.

Crossover probability is selected adaptively as in [35]. Let *f*_max⁡_ be the maximum fitness value of the current population, $\bar{f}$ the average fitness value of the population, and *f*′ the larger of the fitness values of the solutions to be crossed. Then the probability of crossover, *p*_*c*_, is calculated as

(35)  pc=k1×fmax⁡−f′fmax⁡−f¯, if  f′>f¯, pc=k3, if  f′≤f¯.

Here, the values of *k*_1_ and *k*_3_ are equal to 1.0 [35]. Clearly, when $f_{\max}=\bar{f}$ then *f*′ = *f*_max⁡_ and *p*_*c*_ will be equal to *k*_3_. The value of *p*_*c*_ increases when the chromosome is quite poor. In contrast if *p*_*c*_ is low it means chromosome is good. It will prevent the proposed MOLGC algorithm from getting stuck at local optimum.

#### 4.3.3. Mutation

Each cluster center in a chromosome is changed with a random variable generated from a Laplacian distribution [18]. This distribution is characterized by location *μ* (any real number) and scale *δ* parameters. The probability density function of Laplace (*a*, *b*) is

(36) $$\begin{array}{c} p\left(x\right)=\frac{1}{2b}e^{-\left|x-a\right|/b}, \end{array}$$

where the scaling parameter *b* sets the magnitude of perturbation that is referred to as the diversity. Here, parameter *a* denotes the location value which is to be perturbed. We set the scaling parameter *b* equal to 1.0 in our experimental results. In this mutation operation the old value at the mutation position of a chromosome is replaced with newly generated random value using Laplace distribution. The mutation operation is applied for all dimensions of data set independently. The mutation probability *p*_*m*_ is selected adaptively for each chromosome as in [35]. The expression is given below:

(37)  pm=k2×fmax⁡−ffmax⁡−f¯, if  f>f¯, pm=k4, if  f≤f¯,

where *k*_2_ and *k*_4_ are equal to process 0.5. The adaptive mutation process assists genetic algorithm to come out of local optimum. When $f_{\max}=\bar{f}$ value decreases then *p*_*c*_ and *p*_*m*_ both will be increased. As a result GA will come out of local optimum. It will also happen for the global optimum and may result in interference of the near optimal solutions. As a result genetic algorithm never converges to the global optimum. But the values of adaptive crossover probability *p*_*c*_ and adaptive mutation probability *p*_*m*_ will be higher for low fitness solutions and will get low values for higher fitness solutions. The high fitness solutions aid in convergence of the genetic algorithm and the low fitness solutions prevent the genetic algorithm from getting stuck at a local optimum. It may be possible for a solution with highest fitness value; *p*_*c*_ and *p*_*m*_ are both 0. As a result the best solution is transferred into the next generation without crossover and mutation. For selection operator this may lead to an exponential growth of the solution in the population and may cause premature convergence. To overcome the above problem, a default mutation rate (of 0.01) is kept for every solution in the proposed algorithm MOLGC.

### 4.4. Termination Criterion

The proposed multiobjective clustering algorithm has been executed for a fixed number of generations. The fixed number is supplied by the user for terminating the algorithm. After termination, the algorithm gives the best string of the last generation that provides the solution to the clustering problem.

## 5. Experimental Evaluation

The experiments reported in this section were performed on a 2.0 GHz Core 2 Duo processors with 2 GB of memory. We have tested proposed MOLGC algorithm on both real and synthetic data. The qualities of clustering results are measured by adjusted Rand index. We compared the performance of SBKM, MOCK, and MOLGC algorithms. The source code of SBKM is available on Ref. http://mail.tku.edu.tw/chchou/others/SBKM.rar. The source code for the MOCK algorithm is obtained from Ref. (http://personalpages.manchester.ac.uk/mbs/julia.handl/mock.html). For the purpose of comparison, another multiobjective clustering technique, MOCK, is also executed on the above mentioned data sets with default parameter settings. In order to show the effectiveness of the proposed MOLGC clustering technique over existing symmetry based clustering techniques, a symmetry based *K*-means (SBKM) algorithm is executed on both real and synthetic data.

### 5.1. Parameter Setting

The proper setting of parameters in genetic algorithm is crucial for its good performance. Different parameter values might yield very different results. A good setting for algorithm may give best solution within a reasonable time period. In contrast, a poor setting might cause the algorithm to be executed for a very long time before finding a good solution. Sometimes it may so happen that it is not able to find a good solution at all. Grefenstette [36] has used genetic algorithm to investigate the optimal parameters of genetic algorithms. He has reported the best parameter values for GA; these are population size = 30, number of generations = not specified, crossover rate of 0.9, and mutation rate of 0.01. However, the selection of optimal parameters in GA is domain dependent and relies on the specific application areas. Below we justify how the used parameters are selected in MOLGC.

(1) Population size: Goldberg [37] has theoretically analyzed that the optimal population size increases exponentially and is rather large for even moderate chromosome lengths. It has been shown in [37] that the number of schemata processed effectively is proportional to *n* ^3^, where *n* is the population size. This seems to justify the selection of large population size. However, the larger the population size, the longer the genetic algorithm takes to compute each generation. Motivated by above discussion, we set population size = 50 in our proposed algorithm (MOLGC).
(2) Number of generations: A GA generally converges within a few generations. The pure selection convergence times are *O* (log⁡ *N*) generations, where *N* is the size of the population. Thus GA generally searches fairly quickly. In [37] it is mentioned that for a given adequate population size if some linkage knowledge is incorporated into the chromosomes then it is expected that mixing of good building blocks can take place before convergence. Thus it is important to detect near-uniformity of the population and terminate the GA, before wasting function evaluations on an inefficient, mutation-based search. So we set number of generations = 50 (executing MOLGC further did not improve its performance).
(3) Initialization of population: It is customary to initialize genetic algorithm with a population of random individuals. But sometimes previously known (good) solutions can be used to initialize a fraction of the population and this results in faster convergence of GA. In the proposed MOLGC, after randomly generating the cluster centers, some iterations of *K*-means algorithm are executed to separate the cluster centers as much as possible.
(4) Selection of crossover and mutation probabilities: These are two basic parameters of GA. The crossover operation is performed based on crossover probability (*μ*_*c*_). If *μ*_*c*_ = 0, then child offspring is the same copy of parents. If *μ*_*c*_ > 0, then offspring is result of crossover operation on parents chromosome. If *μ*_*c*_ = 1, then all offspring are made by crossover. Crossover operation is performed so that good fitness value parent chromosomes can be combined in the offspring to result in potentially improved solutions. However, it is good to leave some parts of population to survive for the next generation. Mutation probability (*μ*_*m*_) determines how often parts of a chromosome are mutated. If there is no mutation, offspring is taken after crossover (or copy) without any change. If mutation is performed (i.e., *μ*_*m*_ > 0), a part of a chromosome is changed. If mutation probability is 100%, the whole chromosome is changed; if it is 0%, nothing is changed. Mutation is made to prevent GA from falling into local optima. But it should not occur very often; otherwise GA will change to random search. In MOLGC initially the mutation probability and crossover probability were kept fixed. We obtained good results with combination of *μ*_*c*_ = 0.8 and *μ*_*m*_ = 0.01.

The parameters used for proposed MOLGC algorithm in our experimental study are given in Table 1. Apart from the maximum number of clusters, these parameters are kept constant over the entire range of data sets in our comparison study. In this comparison study, the SBKM algorithm is executed for 50 iterations. The parameter *θ* is chosen equal to 0.18 for all data sets. For MOCK algorithm the total number of generation is kept equal to 50.

In order to evaluate the performance of the proposed multiobjective genetic clustering algorithm more objectively, eight artificial data sets and three real data sets are used.

### 5.2. Artificial Data Sets

The artificial data set-1, data set-2, data set-3, and data set-4 are obtained from [7, 17] and remaining data sets were generated by two data generators (http://personalpages.manchester.ac.uk/mbs/julia.handl/generators.html). These generators permit controlling the size and structure of the generated data sets through parameters, such as number of points and dimensionality of the data set.

**Figure 9:**
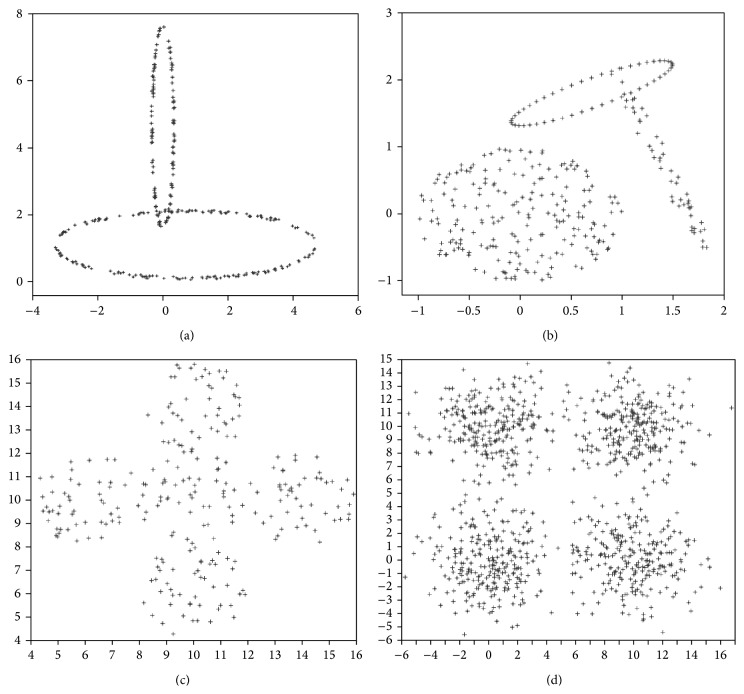
(a) The data set containing two crossed ellipsoidal shells. (b) The data set containing ring shaped, compact, and linear clusters. (c) The data set containing five spherically shaped clusters. (d) The data set containing four linear clusters.

1. Data set-1: This data set, used in [7], contains 300 points distributed on two crossed ellipsoidal shells. This is shown in Figure 9(a).
2. Data set-2: This data set, used in [7], is combination of ring shaped, compact, and linear clusters. The total number of points in it is 300. The dimension of this data set is two. This is shown in Figure 9(b).
3. Data set-3: This data set, used in [7], consists of 250 data points distributed over five spherically shaped clusters. This is shown in Figure 9(c).
4. Data set-4: This data set, used in [17], consists of 1000 data points distributed over four square clusters. This is shown in Figure 9(d).
5. Data set-5: This data set contains 10 dimensional 838 data point's distributed over Gaussian shaped four clusters.
6. Data set-6: This data set consists of 10 dimensional 3050 data points distributed over Gaussian shaped ten clusters.
7. Data set-7: This data set is a 50 dimensional data set and it consists of 351 data points distributed over ellipsoid shaped four clusters.
8. Data set-8: This data set contains 50 dimensional 2328 data points distributed over ellipsoid shaped ten clusters.

The real data sets are obtained from UCI repository (http://archive.ics.uci.edu/ml/). For experimental results four real data sets are considered.

1. Iris: Iris data set consists of 150 data points distributed over three clusters. Each cluster has 50 points. This data set represents different categories of irises characterized by four feature values. It has three classes, Setosa, Versicolor, and Virginica, among which the last two classes have a large amount of overlap while the first class is linearly separable. The sepal area is obtained by multiplying the sepal length by the sepal width and the petal area is calculated in an analogous way.
2. Cancer: Wisconsin breast cancer data set consists of 683 sample points. Each pattern has nine features corresponding to clump thickness, cell size uniformity, cell shape uniformity, marginal adhesion, single epithelial cell size, bare nuclei, bland chromatin, normal nucleoli, and mitoses. There are two categories in the data: malignant and benign. The two classes are known to be linearly separable.
3. Wine: This is the Wine recognition data consisting of 178 instances having 13 features resulting from a chemical analysis of wines grown in the same region in Italy but derived from three different cultivars. The analysis determined the quantities of 13 constituents found in each of the three types of wines.
4. Diabetes: This is the diabetes data set consisting of 768 instances having 8 attributes.

### 5.3. Evaluation of Clustering Quality

To compare the performance of all three algorithms (SBKM, MOCK, and MOGLC) adjusted Rand index technique [38] is used. Let *n*_*lk*_ be the number of objects that are in both class *u*_*l*_ and cluster *v*_*k*_. Let *n*_*l*_ and *n*_*k*_ be the number of objects in class *u*_*l*_ and cluster *v*_*k*_, respectively. Under the generalized hyper geometric model, it can be shown that

(38) $$\begin{array}{c} E\left[\sum_{l,k}\left(\begin{array}{c} n_{lk}\\ 2 \end{array}\right)\right]=\frac{\left[\sum_{l}\left(\begin{array}{c} n_{l.}\\ 2 \end{array}\right)\cdot \sum_{k}\left(\begin{array}{c} n_{k.}\\ 2 \end{array}\right)\right]}{\left(\begin{array}{c} n\\ 2 \end{array}\right)}. \end{array}$$

The adjusted Rand index [38] can be simplified to

(39) $$\begin{array}{c} \frac{\sum_{l,k}\left(\begin{array}{c} n_{lk}\\ 2 \end{array}\right)-\left[\sum_{l}\left(\begin{array}{c} n_{l.}\\ 2 \end{array}\right)\cdot \sum_{k}\left(\begin{array}{c} n_{k.}\\ 2 \end{array}\right)\right]/\left(\begin{array}{c} n\\ 2 \end{array}\right)}{\left(1/2\right)\left[\sum_{l}\left(\begin{array}{c} n_{l.}\\ 2 \end{array}\right)+\sum_{k}\left(\begin{array}{c} n_{k.}\\ 2 \end{array}\right)\right]-\left[\sum_{l}\left(\begin{array}{c} n_{l.}\\ 2 \end{array}\right)\cdot \sum_{k}\left(\begin{array}{c} n_{k.}\\ 2 \end{array}\right)\right]/\left(\begin{array}{c} n\\ 2 \end{array}\right)}. \end{array}$$

Adjusted Rand index is limited to the interval [0,1] with a value of 1 with a perfect clustering. The high value of adjusted Rand index indicates the good quality of clustering result. The average and standard deviation of adjusted Rand index for data sets produced by 20 consecutive runs of SBKM, MOCK, and MOLGC are depicted in Tables 2(a) and 2(b), respectively.

### 5.4. Results on Artificial Data Sets

**Figure 10:**
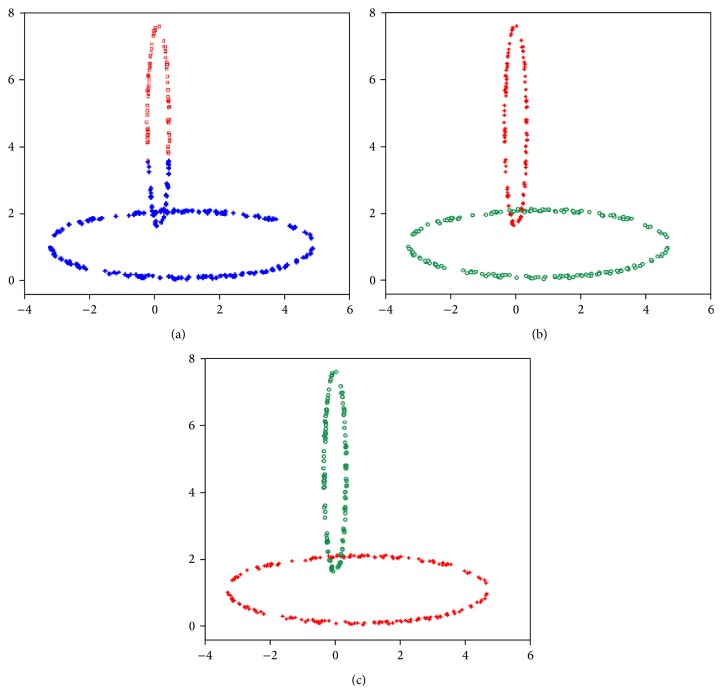
(a) The clustering result achieved by the SBKM (data set-1). (b) The clustering result achieved by the MOCK (data set-1). (c) The clustering result achieved by the MOLGC (data set-1).

**Figure 11:**
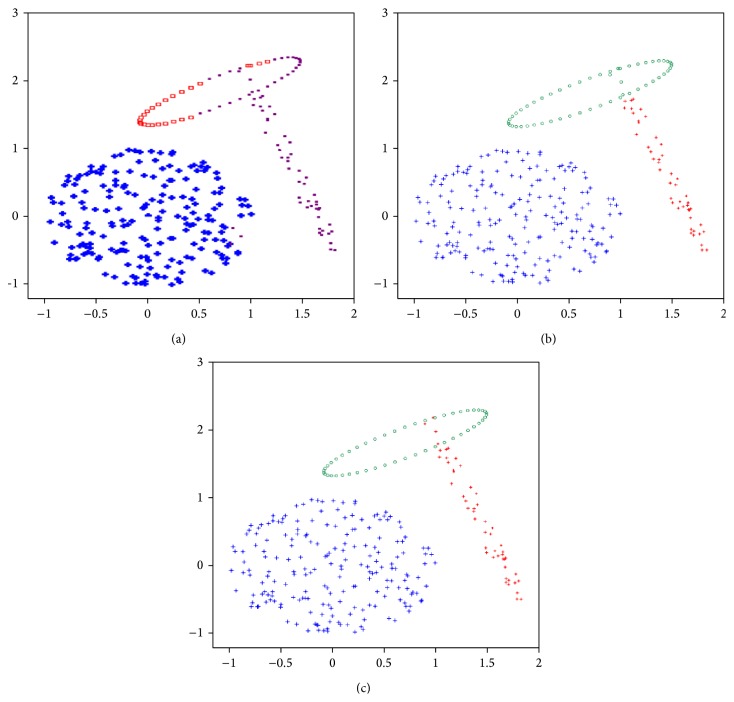
(a) The clustering result achieved by the SBKM (data set-2). (b) The clustering result achieved by the MOCK (data set-2). (c) The clustering result achieved by the MOLGC (data set-2).

**Figure 12:**
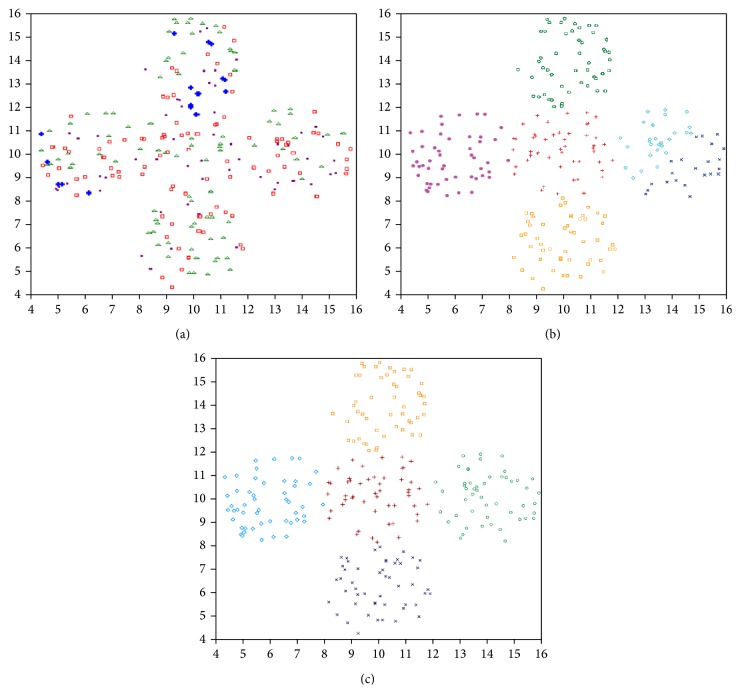
(a) The clustering result achieved by the SBKM (data set-3). (b) The clustering result achieved by the MOCK (data set-3). (c) The clustering result achieved by the MOLGC (data set-3).

**Figure 13:**
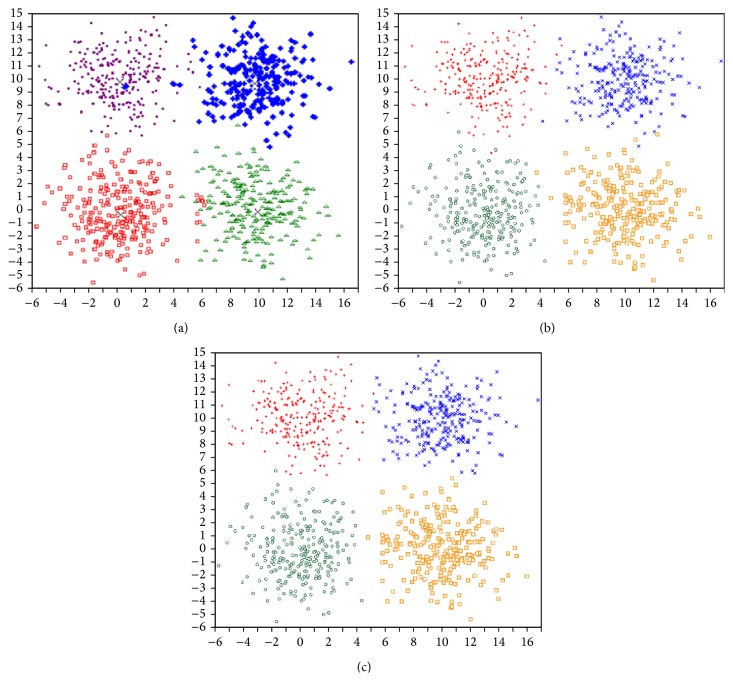
(a) The clustering result achieved by the SBKM (data set-4). (b) The clustering result achieved by the MOCK (data set-4). (c) The clustering result achieved by the MOLGC (data set-4).

(1) Data set-1: We use this data set to illustrate that the proposed algorithm incorporated with line symmetry distance can also be applied to detect ring-shaped clusters even if they are crossed.  Figure 10(a) shows the clustering result achieved by the SBKM algorithm.  Figure 10(b) illustrates the final result achieved by the MOCK algorithm.  Figure 10(c) shows the clustering result of the MOLGC algorithm. We find that the SBKM algorithm cannot work well for this case. Both MOLGC and MOCK clustering algorithms provide *K* = 2 as the optimal number of clusters in different runs. SBKM clustering algorithm discovers *K* = 2 number of clusters but it is unable to perform the proper partitioning from this data set in different runs.
(2) Data set-2: This data set is a combination of ring-shaped, compact, and linear clusters, as shown in Figure 9(b). Most clustering algorithms based on objective function minimization fail to detect this kind of data sets because their performance depends on the dissimilarity measures used to generate a partition of the data sets. The clustering result achieved by the SBKM algorithm is shown in Figure 11(a). The final clustering result of the MOCK algorithm is illustrated in Figure 11(b).  Figure 11(c) shows that the proposed algorithm works well for a set of clusters of different geometrical structures. Both SBKM and MOCK clustering algorithms provide *K* = 3 number of clusters in different runs but both are unable to perform the proper partitioning from this data set. MOLGC clustering algorithm detects *K* = 3 the optimal number of clusters and the proper partitioning from data set-2 in all consecutive runs.
(3) Data set-3: As can be seen from Figure 12(a) to Figure 12(c), for this data set the SBKM clustering technique is unable to detect appropriate number of clusters. The best solution provided by MOCK is not able to determine the appropriate number of clusters from this data set. The corresponding partitioning is shown in Figure 12(b). MOLGC algorithm is able to detect *K* = 5 the appropriate number of clusters from this data set in different runs. The corresponding partitioning is shown in Figure 12(c). MOCK splits data points of one cluster into two clusters and provides *K* = 6 as the optimal number of clusters in different runs. SBKM merges the all data points into four clusters and provides *K* = 4 as the appropriate number of clusters.
(4) Data set-4: Both MOCK and MOLGC clustering algorithms are able to detect *K* = 4 the appropriate number of clusters from this data set in different runs. The clustering result obtained by the SBKM algorithm is shown in Figure 13(a). The partitioning identified by MOCK clustering algorithm is shown in Figure 13(b).  Figure 13(c) shows that the proposed algorithm works well for this data set. SBKM again overlaps the data points in two clusters and discovers *K* = 4 as the optimal number of clusters. It is unable to perform the proper partitioning from this data set in different runs.
(5) Data set-5: The proposed MOLGC algorithm and MOCK algorithms are able to detect *K* = 4 the appropriate number of clusters from this data set in different runs. MOCK merges the data points of two clusters and it is not able to detect proper partitioning from this data set in all runs. SBKM is not able to detect *K* = 5 the appropriate number of clusters and the proper partitioning from this data set in different runs. It again splits data points of one cluster into two clusters and provides *K* = 5 clusters. As shown in the Tables 2(a) and 2(b), the SBKM algorithm cannot work well.
(6) Data set-6: From Tables 2(a) and 2(b), it is clear that proposed MOLGC and MOCK algorithms perform much better on this data set than the other algorithm SBKM. SBKM detects *K* = 12 clusters from this data set. It is unable to provide the appropriate number of clusters and the proper partitioning in different runs. Both MOCK and MOLGC clustering algorithms detect *K* = 10 the appropriate number of clusters from data set-6 in all runs. But MOCK performs overlapping on some data points into two clusters from this data set.
(7) Data set-7: As can be seen from Table 2(a), it is noticeable that MOLGC performs the best (providing the highest adjusted Rand index value) for this data set. The performance of MOCK is also better when compared to SBKM algorithm. For this data set, SBKM provides *K* = 6 as the optimal number of clusters. It splits the maximum dense clusters into two clusters and overestimates the number of clusters from this data set. Both MOCK and MOLGC clustering algorithms produce *K* = 4 the proper number of clusters and partitioning from this data set in different runs. But adjusted Rand index value corresponding to the partitioning obtained by MOLGC is higher than that of MOCK (as shown in Table 2(a)).
(8) Data set-8: As shown in Table 2(a), the adjusted Rand index of MOLGC is the highest for this data set, while the performance of MOCK is second. However, the performance of SBKM algorithm is found poor. For this data set, both SBKM and MOCK detect *K* = 11 as the appropriate number of clusters but both clustering algorithms are unable to produce the appropriate partitioning from this data set in all consecutive runs. The adjusted Rand index values reported in Tables 2(a) and 2(b) also show the poorer performance of both SBKM and MOCK algorithms from this data set. MOLGC discovers *K* = 10 as appropriate number of clusters and the appropriate partitioning from this data set in different runs.

### 5.5. Results on Real Data Sets

(1) Iris: As seen from Table 2(a), the adjusted Rand index of MOLGC is the best for Iris, while the performance of MOCK is second. However, it can be seen from Tables 2(a) and 2(b) that the performance of SBKM algorithm is found poor. SBKM, MOCK, and MOLGC provide *K* = 3 as the appropriate number of clusters form this data set in all consecutive runs. But SBKM detects overlapping of data points in two clusters whereas the third cluster is well separated from these two clusters.
(2) Cancer: As can be seen from Table 2(a), it is manifest that MOLGC performs the best (providing the highest adjusted Rand index value) for this data set. The performance of MOCK and MOLGC is similar, but the performance of SBKM algorithm is found poor. All clustering algorithms are able to provide *K* = 2 the proper number of clusters from this data set in different consecutive runs.
(3) Wine: From Tables 2(a) and 2(b), it is evident that MOLGC performs the best for this data set. Both MOLGC and MOCK clustering algorithms are able to provide *K* = 3 as the proper number of clusters from this data set. The adjusted Rand index value obtained by MOLGC is also the maximum (refer Table 2(a)). SBKM is not able to perform the proper partitioning from this data set.
(4) Diabetes: From Tables 2(a) and 2(b), it is again clear that MOLGC performs much better than the other two algorithms (providing the highest adjusted Rand index value). MOLGC and MOCK clustering algorithms detect *K* = 2 as the optimal number of clusters from this data set. Both clustering algorithms are able to provide the proper partitioning from this data set in different consecutive runs. SBKM is not able to detect appropriate number of clusters in all consecutive runs. The corresponding adjusted Rand index value is reported in Tables 2(a) and 2(b).

It can be seen from the above results that the proposed MOLGC clustering algorithm is able to detect the appropriate number of clusters from most of the data sets used here for the experiments. The superiority of MOLGC is also established on four real-life data sets which are of different characteristics with the number of dimensions varying from 2 to 13. Results on the eight artificial and four real-life data sets establish the fact that MOLGC is well-suited to detect the number of clusters from data sets having clusters of widely varying characteristics.

The performance results reported in Tables 2(a) and 2(b) clearly demonstrate the clustering accuracy of SBKM, MOCK, and MOLGC for artificial and real data sets.  Table 3 indicates average computing time taken by 20 consecutive runs of SBKM, MOCK, and MOLGC for clustering of the above data sets. Results show SBKM and MOCK execution time is increased linearly with increasing dimensions of data sets. The MOLGC shows better results in terms of reduction in CPU time in comparison to SBKM and MOCK.

The proposed MOLGC clustering algorithm is able to identify automatically the appropriate number of clusters in different runs. MOLGC generates the entire set of solutions with automatic determination of correct number of clusters in a single run. It consistently generates the proper cluster number from eight artificial and four real data sets in different runs.

### 5.6. Reconstruction Criterion

In this paper a reconstruction criterion is used to optimize the performance of the SBKM, MOCK, and MOLGC clustering algorithms. A fuzzy C-means (FCM) algorithm based clustering platform is considered in [39]. The objective of this work is to raise awareness about the essence of the encoding and decoding processes completed in the context of fuzzy sets. The main design aspects deal with the relationships between the number of clusters and the reconstruction properties and the resulting reconstruction error. Let *X* = {*x*_1_, *x*_2_,…, *x*_*n*_} be a set of *n* points in a multidimensional experimental data set. Now three sets of prototypes (*v*_1_, *v*_2_,…, *v*_*c*_) are generated by running the SBKM, MOCK, and MOLGC clustering algorithms separately on experimental data. For any data point *x*_*i*_, we obtain its membership grades to the corresponding clusters. They are denoted by *u*_*ij*_ (*i* = 1,2,…, *c* and *j* = 1,2,…, *n*) and are result of the minimization of the following objective function:

(40) $$\begin{array}{c} \overset{c}{\underset{i=1}{\sum }}\;\overset{n}{\underset{j=1}{\sum }}u_{ij}^{m}d^{2}\left(v_{i},v_{j}\right), \end{array}$$

where *m*  (*m* > 1) is a coefficient. The distance *d* used in the objective function is viewed as the Point Symmetry Distance (PSD) in SBKM [7], nearest neighbor consistency (MOLGC), and line symmetry distance in MOLGC:

(41) $$\begin{array}{c} \overset{c}{\underset{i=1}{\sum }}\;\overset{n}{\underset{j=1}{\sum }}u_{ij}=1. \end{array}$$

By solving (41) through the use of Lagrange multipliers, we arrive at the expression of the granular representation of the numeric value:

(42) $$\begin{array}{c} u_{ij}=\frac{1}{\sum_{k=1}^{c}{\left(d\left(v_{i},x_{j}\right)/d\left(v_{k},x_{j}\right)\right)}^{2/\left(m-1\right)}}. \end{array}$$

**Figure 14:**
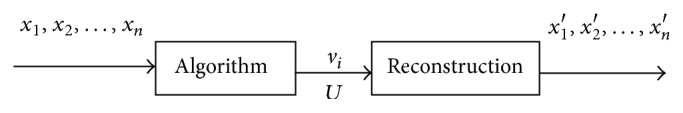
Scheme of the reconstruction criterion.

Figure 14 highlights the essence of reconstruction criterion. Our starting point is the result of clustering expressed in terms of the prototypes and the partition matrix.

The main objective of this reconstruction process is to reconstruct the original data using the cluster prototypes and the partition matrix by minimizing the sum of distances [39]:

(43) $$\begin{array}{c} F=\overset{c}{\underset{i=1}{\sum }}\;\overset{n}{\underset{j=1}{\sum }}u_{ij}^{m}d^{2}\left(v_{i},x_{j}^{\prime }\right), \end{array}$$

where *x*_*j*_′ is the reconstructed version of *x*_*j*_. We used the Point Symmetry Distance (PSD) for SBKM [7], nearest neighbor consistency for MOCK, and line symmetry distance for MOLGC in (43) and zeroing the gradient of *F* with respect to *x*_*k*_, we have

(44) $$\begin{array}{c} x_{j}^{\prime }=\frac{\sum_{i=1}^{c}u_{ij}^{m}v_{i}}{\sum_{i=1}^{c}u_{ij}^{m}}. \end{array}$$

The performance of reconstruction is expressed as

(45) $$\begin{array}{c} E=\overset{n}{\underset{J=1}{\sum }}{\left\|x_{j}-x_{j}^{\prime }\right\|}^{2}. \end{array}$$

We investigate the behavior of the clustering results quantified in terms of the criteria of reconstruction for artificial and real data sets.  Table 4 presents the reconstruction error values reported for clusters by 20 consecutive runs of SBKM, MOCK, and MOLGC, respectively. In all experiments, the value of the coefficient *m* was set to 2.

### 5.7. Statistical Significance Test

For a more careful comparison among SBKM, MOCK, and MOLGC, a statistical significance test called Wilcoxon rank sum test [40] for independent samples has been conducted at the 5% significance level. It is a nonparametric alternative to the paired *t*-test. It assumes commensurability of differences, but only qualitatively: greater differences still count more, which is probably desired, but the absolute magnitudes are ignored. From the statistical point of view, the test is safer since it does not assume normal distributions. Also, the outliers have less effect on the Wilcoxon test than on the *t*-test. The Wilcoxon test assumes continuous differences *d*_*i*_; therefore they should not be rounded to, say, one or two decimals since this would decrease the power of the test due to a high number of ties. When the assumptions of the paired *t*-test are met, the Wilcoxon rank test is less powerful than the paired *t*-test. On the other hand, when the assumptions are violated, the Wilcoxon test can be even more powerful than the *t*-test. Three groups corresponding to three algorithms SBKM, MOCK, and MOLGC have been created for each data set. Each group consists of the performance scores (adjusted Rand index for the artificial data and real life data) produced by 20 consecutive runs of corresponding algorithm. The median values of each group for all the data sets are shown in Table 5. The results obtained with this statistical test are shown in Table 6. To establish that this goodness is statistically significant, Table 6 reports the *P* values produced by Wilcoxon's rank sum test for comparison of groups (SBKM, MOCK, and MOLGC) at a time. As a null hypothesis, it is assumed that there are no significant differences between the median values of two groups. However, the alternative hypothesis is that there is significant difference in the median values of the two groups. All the *P* values reported in the table are less than 0.05 (5% significance level).

The smaller the *P* value, the stronger the evidence against the null hypothesis provided by the data. The signed rank test among algorithms MOLGC, SBKM, and MOCK for artificial data and real life data provides a *P* value, which is very small. This is strong evidence against the null hypothesis, indicating that the better median values of the performance metrics produced by MOLGC are statistically significant and have not occurred by chance. Similar results are obtained for all other data sets and for all other algorithms compared to MOLGC, establishing the significant superiority of the MOLGC algorithm.

## 6. Conclusion

In this paper, a line symmetry based multiobjective MOLGC algorithm is proposed. In the proposed algorithm, the points are assigned to different clusters based on line symmetry based distance. In this multiobjective genetic clustering algorithm two objective functions, one based on a new line symmetry based distance and another based on Euclidean distance DB index, are used for computation of fitness. The proposed algorithm can be used to group given data set into a set of clusters of different geometrical structures. Compared with the SBKM and the MOCK, the proposed MOLGC algorithm adopts a line symmetry approach to cluster data; therefore, the later approach is more flexible. Most importantly, a modified version of the line symmetry distance is proposed to overcome some limitations of the original version of the symmetry distance introduced by Chung and Lin [8]. In addition, the MOLGC algorithm outperforms the SBKM algorithm and the MOCK based on the comparisons of the results presented in this paper. Tables 2(a) and 2(b) indicate the quality of best clustering results in terms of adjusted Rand index generated by SBKM, MOCK, and MOLGC for eight artificial data sets and four real data sets.  Table 3 tabulates the comparisons of the computational time of the MOLGC algorithm and other popular clustering algorithms. Obviously, the proposed algorithm needs more computational resources than other algorithms. However, the proposed algorithm provides a possible solution to detect clusters with a combination of compact clusters, shell clusters, and line-shaped clusters. It should be emphasized that although the present MOLGC algorithm demonstrates to some extent the potential of detecting clusters with different geometrical structures, there still remains a lot of research space for improving the MOLGC algorithm, such as how to reduce the computational time.

Finally, it is an interesting future research topic to extend the results of this to face recognition.

## Figures and Tables

**Algorithm 1:**
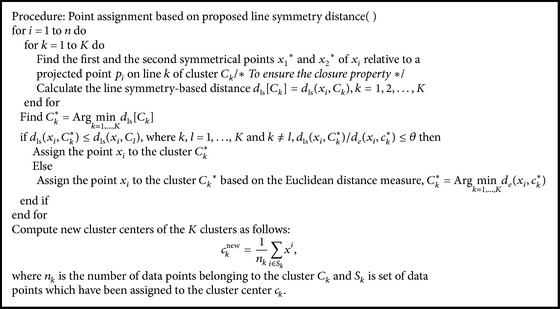
Point assignment based on proposed line symmetry distance.

**Algorithm 2:**
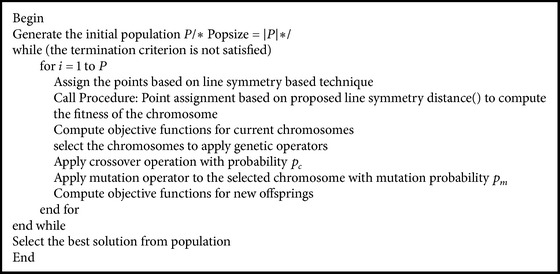
Steps of proposed algorithm.

**Algorithm 3:**
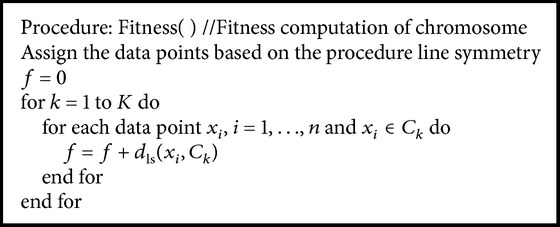
The procedure of the fitness computation.

**Table 1:** Parameter setting for proposed MOLGC algorithm.

Parameter	Setting
Number of generations	50
Population size	50
Number of clusters	*K*_min⁡_ to *K*_max⁡_ [2 to 20]
Crossover probability (*p*_*c*_)	0.8
Mutation probability (*p*_*m*_)	0.01

**(a):** 

Data sets	Number of points	Number of dimensions	Number of clusters	Average value of adjusted Rand index		
	(*N*)	(*D*)	(*K*)	SBKM	MOCK	MOLGC
Data set-1	200	2	2	0.7585	0.9840	0.9845
Data set-2	350	2	3	0.7491	0.9245	0.9515
Data set-3	250	2	5	0.5158	0.9510	0.9910
Data set-4	1000	2	4	0.8605	0.9815	0.9816
Data set-5	838	10	4	0.7225	0.9862	0.9895
Data set-6	3050	10	10	0.6585	0.9673	0.9795
Data set-7	351	50	4	0.6775	1.0000	1.0000
Data set-8	2328	50	10	0.6325	0.9950	0.9955
Iris	150	4	3	0.7685	0.9350	0.9810
Cancer	683	9	2	0.7877	0.9520	0.9740
Wine	178	13	3	0.6591	0.9575	0.9585
Diabetes	768	8	2	0.7111	0.9840	0.9910

**(b):** 

Data sets	Number of points	Number of dimensions	Number of clusters	Standard deviation of adjusted Rand index		
	(*N*)	(*D*)	(*K*)	SBKM	MOCK	MOLGC
Data set-1	200	2	2	0.075	0.040	0.035
Data set-2	350	2	3	0.081	0.055	0.045
Data set-3	250	2	5	0.090	0.078	0.050
Data set-4	1000	2	4	0.121	0.095	0.055
Data set-5	838	10	4	0.125	0.085	0.060
Data set-6	3050	10	10	0.150	0.070	0.028
Data set-7	351	50	4	0.175	0.075	0.041
Data set-8	2328	50	10	0.190	0.090	0.035
Iris	150	4	3	0.080	0.036	0.022
Cancer	683	9	2	0.090	0.045	0.037
Wine	178	13	3	0.085	0.051	0.035
Diabetes	768	8	2	0.070	0.050	0.030

**Table 3:** Comparison of the execution time (in seconds).

Data sets	SBKM	MOCK	MOLGC
Data set-1	10	15	18
Data set-2	12	18	20
Data set-3	25	35	40
Data set-4	40	45	48
Data set-5	70	52	50
Data set-6	1450	378	365
Data set-7	246	156	150
Data set-8	5300	656	640
Iris	10	14	14
Cancer	62	45	42
Wine	55	30	28
Diabetes	60	35	32

**Table 4:** Reconstruction error for the artificial and real data sets.

Data set	Cluster value	SBKM	MOCK	MOLGC
Data set-1	2	19.50	14.35	11.45
Data set-2	3	17.15	13.72	10.25
Data set-3	5	15.45	10.45	9.14
Data set-4	4	16.15	11.25	9.45
Data set-5	4	17.12	12.25	10.50
Data set-6	10	11.15	8.50	5.25
Data set-7	4	17.10	12.35	8.06
Data set-8	10	12.50	8.55	3.85
Iris	3	15.82	12.47	9.25
Cancer	2	18.45	13.25	10.15
Wine	3	17.35	13.53	8.97
Diabetes	2	19.10	11.72	9.55

**Table 5:** Median values of adjusted Rand index for artificial and real data sets.

Data sets	SBKM	MOCK	MOLGC
Data set-1	0.7599	0.9880	0.9895
Data set-2	0.7510	0.9297	0.9555
Data set-3	0.5199	0.9560	0.9940
Data set-4	0.8685	0.9875	0.9850
Data set-5	0.7280	0.9885	0.9915
Data set-6	0.6625	0.9690	0.9825
Data set-7	0.6805	1.0000	1.0000
Data set-8	0.6375	0.9915	0.9980
Iris	0.7715	0.9380	0.9875
Cancer	0.7901	0.9565	0.9780
Wine	0.6610	0.9605	0.9615
Diabetes	0.7157	0.9870	0.9925

**Table 6:** *P* values produced by Wilcoxon Rank test for comparing MOLGC with SBKM and MOCK.

Data sets	*P* values	
	SBKM	MOCK
Data set-1	1.53*E* − 4	1.65*E* − 4
Data set-2	1.48*E* − 4	1.70*E* − 4
Data set-3	1.70*E* − 3	1.21*E* − 4
Data set-4	1.10*E* − 4	5.14*E* − 5
Data set-5	2.28*E* − 3	1.55*E* − 4
Data set-6	2.45*E* − 3	6.55*E* − 5
Data set-7	1.19*E* − 4	1.85*E* − 5
Data set-8	1.41*E* − 4	2.25*E* − 5
Iris	2.31*E* − 4	1.32*E* − 4
Cancer	1.35*E* − 5	1.65*E* − 5
Wine	1.20*E* − 4	5.75*E* − 5
Diabetes	1.30*E* − 4	1.25*E* − 4
